# Ketones Elicit Distinct Alterations in Adipose Mitochondrial Bioenergetics

**DOI:** 10.3390/ijms21176255

**Published:** 2020-08-29

**Authors:** Chase M. Walton, Samuel M. Jacobsen, Blake W. Dallon, Erin R. Saito, Shantelle L. H. Bennett, Lance E. Davidson, David M. Thomson, Robert D. Hyldahl, Benjamin T. Bikman

**Affiliations:** 1 Department of Physiology and Developmental Biology, Brigham Young University, Provo, UT 84602, USA; chase.m.walton@gmail.com (C.M.W.); sam.jacobsen@icloud.com (S.M.J.); bdallon@gmail.com (B.W.D.); ersaito3@gmail.com (E.R.S.); lenaehouse@gmail.com (S.L.H.B.); david_thomson@byu.edu (D.M.T.); 2 Department of Exercise Sciences, Brigham Young University, Provo, UT 84602, USA; lance.davidson@byu.edu (L.E.D.); robhyldahl@byu.edu (R.D.H.)

**Keywords:** mitochondria, uncoupling, ketones, adipocyte

## Abstract

Objective: The rampant growth of obesity worldwide has stimulated explosive research into human metabolism. Energy expenditure has been shown to be altered by diets differing in macronutrient composition, with low-carbohydrate, ketogenic diets eliciting a significant increase over other interventions. The central aim of this study was to explore the effects of the ketone β-hydroxybutyrate (βHB) on mitochondrial bioenergetics in adipose tissue. Methods: We employed three distinct systems—namely, cell, rodent, and human models. Following exposure to elevated βHB, we obtained adipose tissue to quantify mitochondrial function. Results: In every model, βHB robustly increased mitochondrial respiration, including an increase of roughly 91% in cultured adipocytes, 113% in rodent subcutaneous adipose tissue (SAT), and 128% in human SAT. However, this occurred without a commensurate increase in adipose ATP production. Furthermore, in cultured adipocytes and rodent adipose, we quantified and observed an increase in the gene expression involved in mitochondrial biogenesis and uncoupling status following βHB exposure. Conclusions: In conclusion, βHB increases mitochondrial respiration, but not ATP production, in mammalian adipocytes, indicating altered mitochondrial coupling. These findings may partly explain the increased metabolic rate evident in states of elevated ketones, and may facilitate the development of novel anti-obesity interventions.

## 1. Introduction

In the United States and beyond, obesity has reached a remarkable prevalence. A total of 42% of US adults are obese [1], and an estimated 88% of adults are considered metabolically unhealthy [2]. Understandably, the explosion in obesity, and our ongoing failure to broadly address it, has given rise to intensive efforts to better understand adipose tissue physiology. These efforts have revealed, among other things, that humans store fat in two distinct depots that differ by function: white adipose tissue (WAT) and brown adipose tissue (BAT). Whereas WAT primarily acts to store energy for subsequent use with a very low energy expenditure and low mitochondrial content, BAT is enriched with very active mitochondria that manifest a surprisingly high energy expenditure, serving a thermogenic role. 

In higher vertebrates, WAT acts primarily for lipid storage, centralized in unilocular lipid droplets within adipocytes that are catabolized and released as fatty acids when necessary. However, obesity is typified by excess WAT accumulation, which poses a meaningful risk for developing insulin resistance and type 2 diabetes mellitus [3,4], cardiovascular disease [5], and some cancers [6]. In stark contrast, BAT has a limited lipid storage, with multi-locular lipid droplets co-mingled with a substantial population of mitochondria (giving rise to the distinct reddish-brown color). Increased BAT mass and activity are associated with a resistance to obesity and related disorders [7,8]. Perhaps the most unique aspect of BAT is the nature of its mitochondria—they express a proteome that fosters energy wasting by uncoupling oxidative phosphorylation from electron transport. In contrast, the mitochondria in WAT are very tightly coupled, which is evidence of a more efficient energy storage profile [9].

WAT and BAT rapidly undergo adaptive and dynamic changes in response to both energy and temperature changes [10]. The initial changes within the first 24 h may only involve an altered expression of proteins. However, after 2–3 days, dietary and environmental stimuli can induce marked tissue remodeling, which results in altered adipose tissue morphology and possibly also modified functional properties [11]. In particular, WAT is able to manifest the characteristics of BAT, including mitochondrial biogenesis and uncoupling, a process known as “beiging” [12,13]. Insofar as obesity may partly derive from reduced BAT activity [1], inducing its activity or pushing WAT to manifest BAT characteristics could add a new approach to combating the condition.

While ketones such as acetoacetate and its more prevalent metabolite β-hydroxybutyrate (βHB) are known fuel sources for all cells with mitochondria [14], recent work has detailed their roles as signaling molecules, altering inflammation [15], cognition [16,17,18], oxidative stress [19], and more [20,21]. Moreover, ketones are a critical intermediate between adipose tissue and brain energy fuel supply, allowing the brain to meet energetic requirements during glucose restriction and starvation [22,23,24]. Furthermore, βHB acts as a signal to directly regulate the metabolism and maintain energy homeostasis during nutrient deprivation [25]. 

Perhaps due to both their energy potential and effects on cellular signaling, ketones appear to facilitate a more favorable metabolic milieu that should be considered a tool in our collective efforts to address human obesity [14]. Thus, with the complex mechanistic regulation of adipose physiology in mind, and the role of ketones as metabolic intermediates and signaling molecules, we aimed to explore the effect of the prominent ketone βHB on adipose tissue mitochondrial bioenergetics.

## 2. Results

### 2.1. β-Hydroxybutyrate Alters Mitochondrial Respiration in Cultured Adipocytes

**Figure 1:**
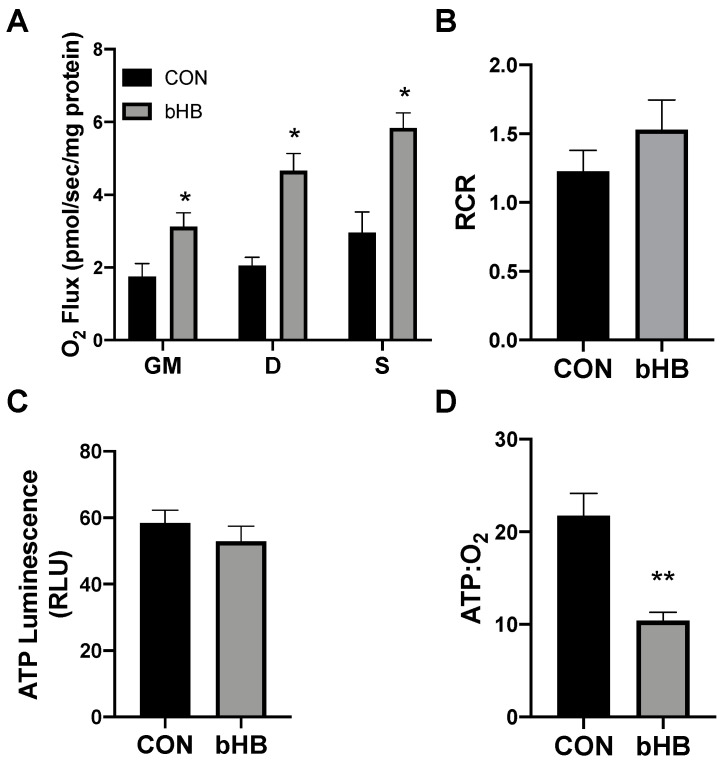
β-hydroxybutyrate (βHB) alters mitochondrial bioenergetics in cultured adipocytes. Following 24-h treatment with βHB (5 mM), the adipocytes were harvested and used to measure the mitochondrial respiration (**A**) and respiratory control ratio (RCR; **B**) in adipocytes. Permeabilized adipocytes were sequentially treated with glutamate (10 mM) and malate (2 mM; GM); +ADP (D; 2.5 mM); +succinate (S; 10 mM). ATP was measured via luminescence from freshly lysed cells (**C**), and the ratio of ATP and respiration was quantified (**D**). *n* = 5. * *p* < 0.05. ** *p* < 0.01.

Following 24 h of treatment with β-hydroxybutyrate (βHB), the adipocytes exhibited an increased rate of respiration (Figure 1A), evident after the addition of ADP (D), and continued with the addition of succinate (S). However, the overall respiratory function of the mitochondria appeared to be similar, as there was no significant difference in the respiratory control ratio (RCR) (Figure 1B). 

Beyond mitochondrial respiration, we sought to better understand the effects of ketones in altering ATP production. Although the analysis of mitochondrial respiration indicated an increased rate of oxygen use, we found that βHB had no significant effect on the ATP production in cultured adipocytes (Figure 1C). The combination of these two variables (i.e., respiration and ATP production) enables an assessment of mitochondrial coupling. Accordingly, we found that βHB treatment resulted in a significant elevation in the ratio of oxygen consumed per unit of ATP produced, revealing an increased uncoupling in βHB-treated adipocytes (Figure 1D).

### 2.2. β-Hydroxybutyrate Changes Adipocyte Gene Expression 

**Figure 2:**
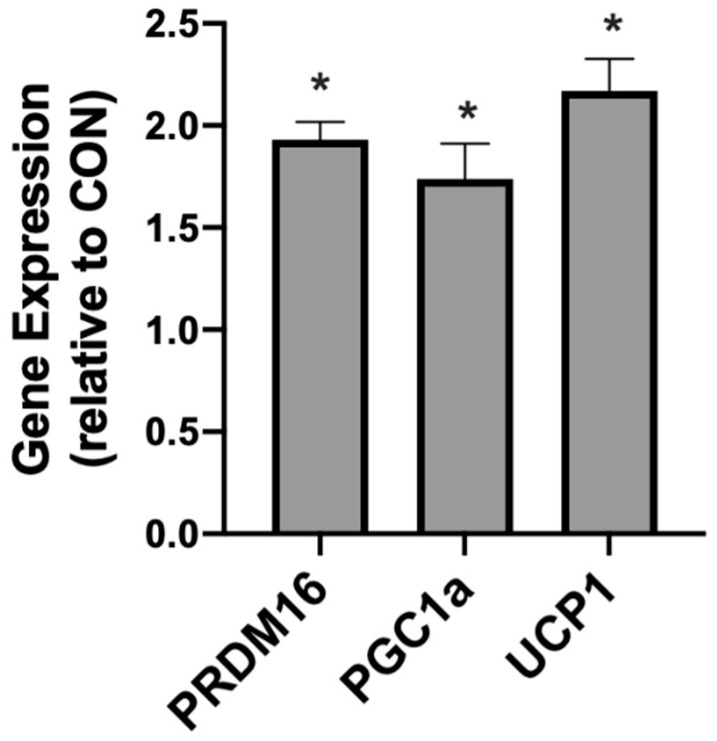
β-hydroxybutyrate (βHB) alters the mitochondrial-related gene expression in cultured adipocytes. Following treatment with βHB (5 mM; 24 h), the cells were harvested and the expression of PRDM16, PGC1α, and UCP1 was quantified. *n* = 6. * *p* < 0.05.

In addition to meaningful changes in mitochondrial uncoupling, the βHB-treated adipocytes responded with an increased expression of genes associated with mitochondrial uncoupling. Namely, we detected significantly higher levels of PRDM16, PGC1α, and UCP1 when compared with the control conditions (Figure 2).

### 2.3. A Ketogenic Diet Alters Adipose Mitochondrial Bioenergetics

**Figure 3:**
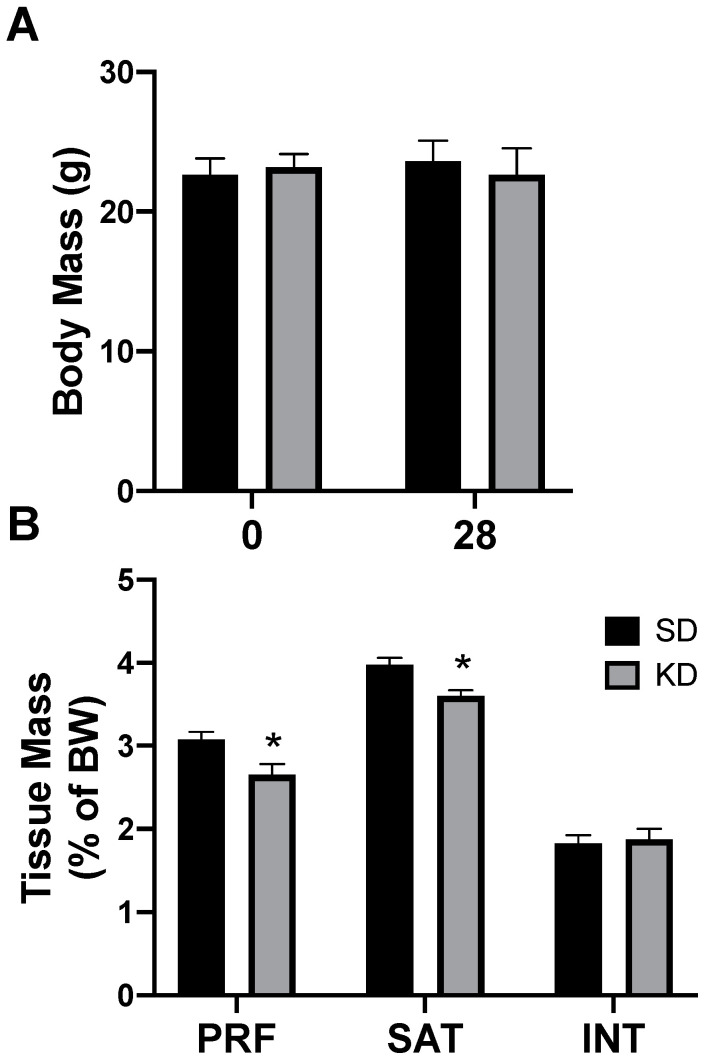
A ketogenic diet selectively alters the fat mass in rodent adipose tissue. Following 28 days on a ketogenic diet (KD) or standard diet (SD), the body mass (**A**) and adipose mass (**B**) from the perirenal (PRF), subcutaneous inguinal (SAT), and interscapular (INT) depots was quantified. *n* = 6. * *p* < 0.05.

Rats had free access to a ketogenic diet (KD) or a standard diet (SD). After two days, and through the remainder of the study, caloric intake was not different between diets, corroborating the results from previous work [26]. Through the 4-week experimental period, there were no significant differences in body weight, regardless of the treatment or time (Figure 3A). Interestingly, only select depots of adipose tissue were affected by the ketogenic diet; the perirenal (PRF) and subcutaneous (SAT) inguinal adipose mass significantly decreased in rats fed a KD, while there was no significant difference in the interscapular (INT) fat (Figure 3B). No differences were noted between the sexes (data not shown). 

**Figure 4:**
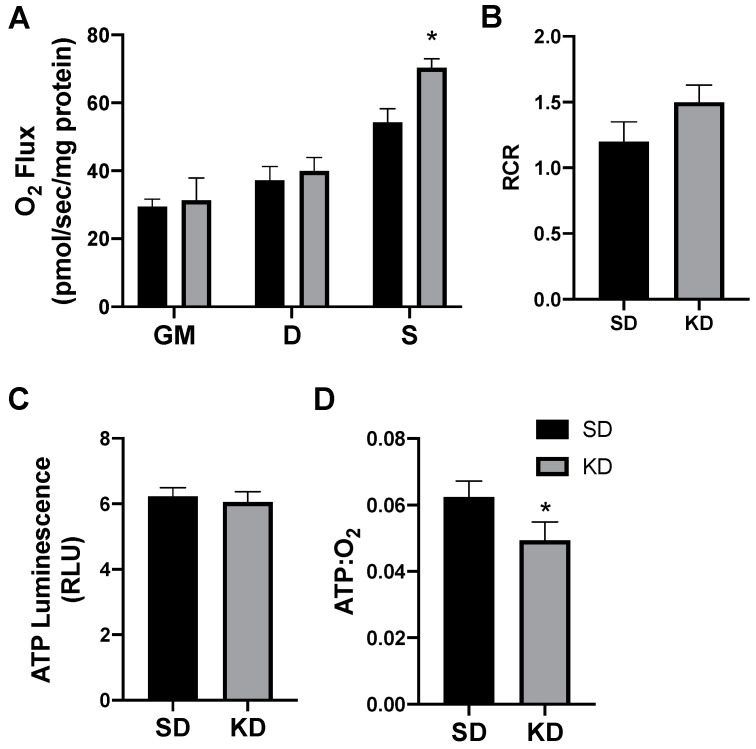
A ketogenic diet minimally alters rodent interscapular adipose mitochondrial bioenergetics. Following the 28-day diet intervention, the interscapular fat was harvested and permeabilized to measure the mitochondrial respiration (**A**) and respiratory control ratio (RCR; **B**). ATP was measured via luminescence from fresh tissue (**C**), and the ratio of ATP and oxygen respiration was quantified (**D**). *n* = 6. * *p* < 0.05.

**Figure 5:**
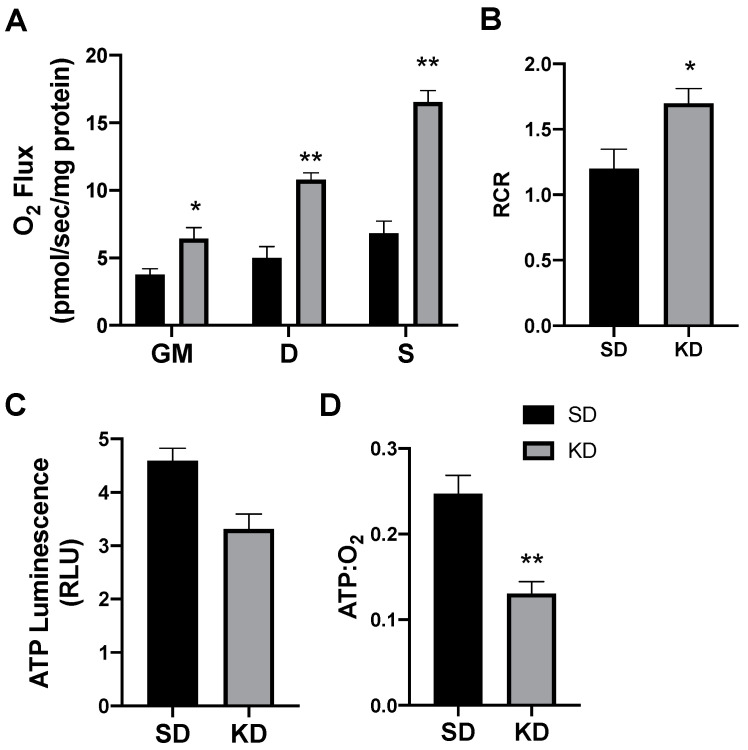
A ketogenic diet alters rodent subcutaneous inguinal adipose mitochondrial bioenergetics. Following the 28-day intervention of either ketogenic diet (KD) or standard diet (SD), the inguinal adipose was harvested and permeabilized to measure the mitochondrial respiration (**A**) and respiratory control ratio (RCR; **B**). ATP was measured via luminescence from fresh tissue (**C**), and the ratio of ATP and oxygen respiration was quantified (**D**). *n* = 6. * *p* < 0.05. ** *p* < 0.01.

**Figure 6:**
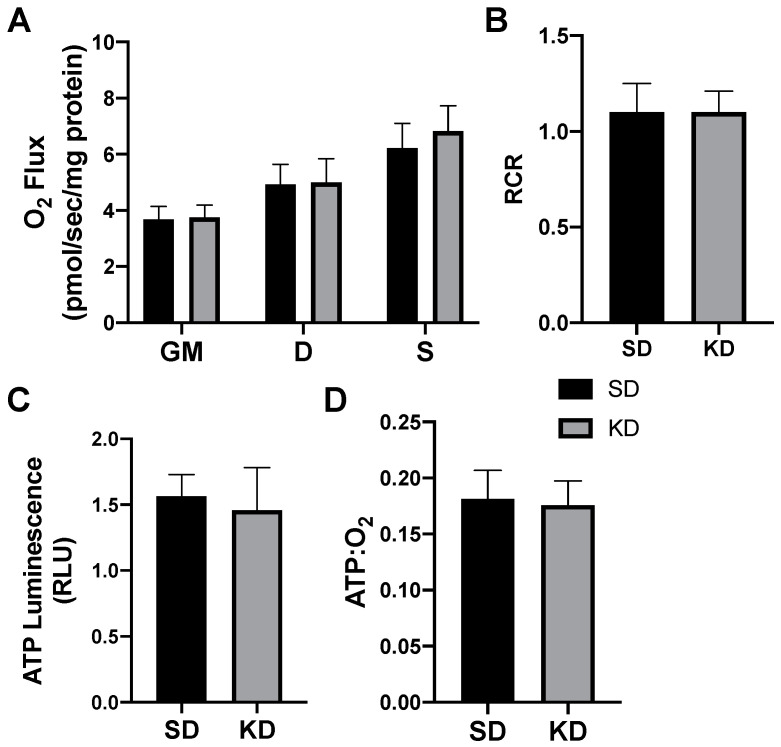
A ketogenic diet does not alter the rodent perirenal adipose mitochondrial bioenergetics. Following the 28-day ketogenic (KD) or standard diet (SD) intervention, perirenal fat was harvested and permeabilized to measure the mitochondrial respiration (**A**) and respiratory control ratio (RCR; **B**). ATP was measured via luminescence from fresh tissue (**C**), and the ratio of ATP and oxygen respiration was quantified (**D**). *n* = 6.

In rodents fed a KD, the INT mitochondrial respiration was slightly increased (Figure 4A,B). The ATP generation in INT (Figure 4C) was similar between the treatments, but KD reduced the ratio of ATP generated to oxygen consumed (Figure 4D). SAT had widespread significant elevations in the mitochondrial respiration rate (Figure 5A) and respiratory control ratio (RCR; Figure 5B). However, the ATP generation was not significantly changed compared with fat from standard chow-fed controls (CON; Figure 5C). Accordingly, the ratio of ATP produced compared with oxygen consumed was significantly lower in the KD tissue (Figure 5D). In contrast, the PRF did not express any significant difference between the SD and KD groups in respiration (Figure 6A), RCR (Figure 6B), ATP generation (Figure 6C), or the ATP:O_2_ ratio (Figure 6D). 

### 2.4. A Ketogenic Diet Alters Adipose Tissue Genetics in a Depot-Specific Manner

**Figure 7:**
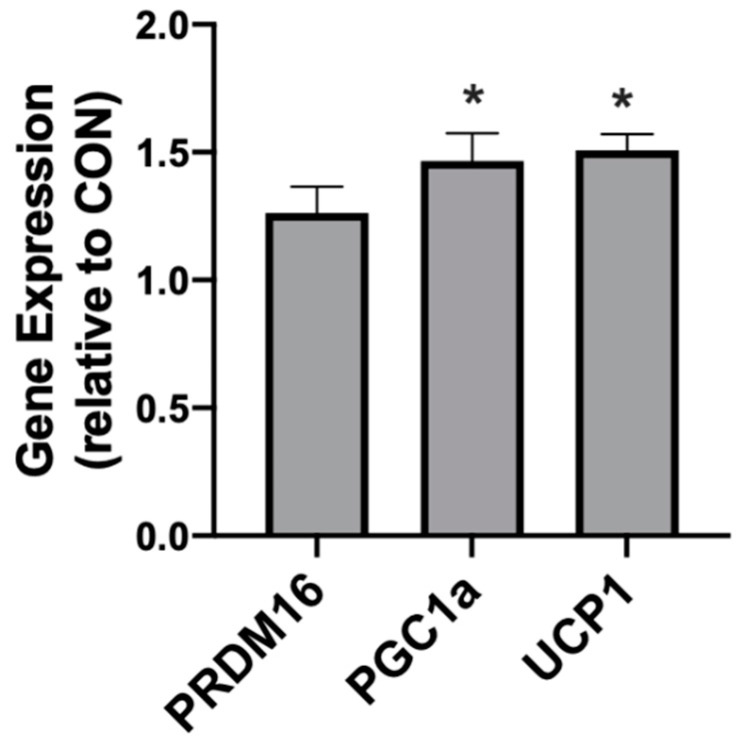
A ketogenic diet alters the mitochondrial-related gene expression in rodent subcutaneous adipose. Following the 28-day diet intervention, subcutaneous adipose was harvested and the expression of PRDM16, PGC1α, and UCP1 was quantified. *n* = 6. * *p* < 0.05.

Following 4 weeks of diet intervention, the three adipose depots (PRF, SAT, INT) exhibited different responses in genes associated with browning and mitochondrial uncoupling. Neither the INT or PRF depots expressed any significant difference between the treatments in PGC1α, PRDM16, or UCP1. However, in KD-fed rats the SAT expressed significantly higher levels of PGC1α and UCP1, but not PRDM16 (Figure 7).

### 2.5. Shifts in Ketone-Induced Mitochondrial Bioenergetics Are Conserved in Human Adipose 

**Figure 8:**
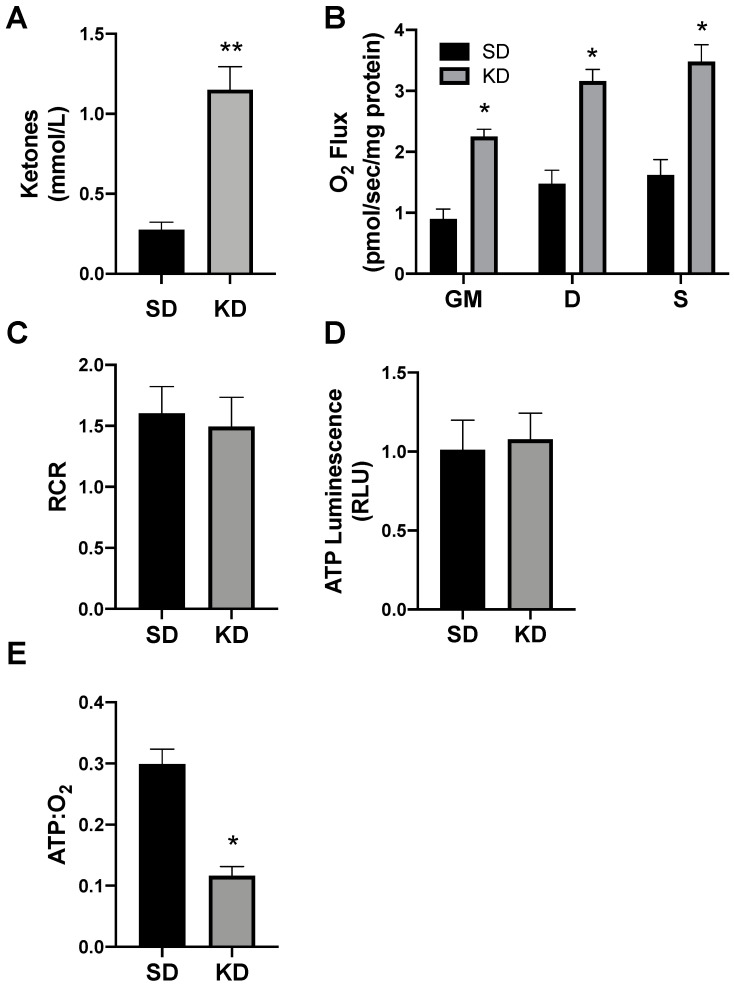
Ketosis alters human subcutaneous adipose mitochondrial bioenergetics. A subcutaneous adipose biopsy was performed in adults adhering to a standard American diet (SD; *n* = 4) or ketogenic diet (KD; *n* = 5). Ketosis was confirmed via plasma ketone measurements (**A**). Following the adipose biopsy, tissue was permeabilized to measure the mitochondrial respiration (**B**) and respiratory control ratio (RCR; **C**). ATP was measured via luminescence following respiration (**D**), and the ratio of ATP and oxygen respiration was quantified (**E**). * *p* < 0.05. ** *p* < 0.01.

To determine the proof of concept of this phenomenon in humans, we biopsied subcutaneous adipose (SAT) from volunteers adhering to a standard (SD) or ketogenic diet (KD; Figure 8A). There were no significant differences in the subject characteristics (as outlined in Methods), though age was nearly significant (*p* = 0.07). Similar to SAT from rodents, the SAT from volunteers in ketosis had broad elevations in mitochondrial respiration (Figure 8B), though there was no significant difference in the RCR (Figure 8C). The SAT from people in ketosis revealed comparable levels of ATP production as SAT from SD volunteers (Figure 8D), which, taken altogether, revealed substantially greater adipocyte mitochondrial uncoupling (Figure 8E). 

## 3. Discussion

Across cell, rodent, and human models, the central conclusion from our studies is that ketones stimulate mitochondrial uncoupling in subcutaneous adipose tissue. As with all nutrients, particularly carbohydrate and fat, ketones are both a source of energy and a signaling molecule. However, ketones appear to elicit a unique effect with regard to energy use in adipocytes. Whereas carbohydrate and fat, independent of their caloric value, activate processes to store energy in adipocytes, such as ChREBP [27] and PPARγ [28], respectively, ketones appear to activate processes that spend and even waste energy via mitochondrial uncoupling [29]. 

Our findings of increased mitochondrial uncoupling (i.e., reduced ATP:O_2_) in adipose in response to ketone exposure corroborates previous work from the Veech lab, which identified an effect of ketones enhancing the native mitochondrial uncoupling within brown adipose tissue (BAT) [29]. BAT is found in limited amounts in adult humans, though it can nevertheless be activated via cold exposure and exercise and offer some protection against obesity [30,31]. Far more abundant than BAT is subcutaneous adipose (SAT), and while generally more metabolically inert, SAT has the capacity to both induce mitochondrial biogenesis and uncoupling, making it characteristically more similar to BAT [32]. Though we were unable to quantify UCP1 from our human SAT samples, our evidence of reduced ATP production compared to O_2_ consumption indicates a greater degree of mitochondrial uncoupling in human SAT when in ketosis. A limitation to this conclusion with the human tissue is the method we used to quantify ATP. Whereas ATP was quantified from cultured adipocytes and rodent adipose without permeabilization, due to constraints on human adipose sample mass, we measured the ATP following permeabilization, which could lead to artificially lower ATP levels due to potential loss through the permeabilized cell membrane. However, we found very little difference in ATP levels in cultured adipocytes and rodent tissue before and after permeabilization, which lends confidence to our findings. 

Other studies that have measured mitochondrial coupling have generally utilized one of two methods, either singularly measuring mitochondrial respiration or a combination of respiration with ATP quantification. The former relies on the ATP synthase inhibitor oligomycin. We elected the latter option, as others have done [5,6], which we consider a more explicit indicator of the degree of synchronization between electron transport (i.e., respiration) and ATP generation (i.e., oxidative phosphorylation). Because we sought to make a direct comparison of oxygen use to ATP generation, and did not base our findings on respiration alone, we did not use oligomycin in our respiration protocols. 

Visceral and subcutaneous WAT both contain the potential towards increased mitochondrial uncoupling, though each appears to respond to unique stimuli. While SAT responds to nitric oxide [7], visceral adipose responds to catecholamines [8]. Based on our rodent work, ketones appear to be a unique stimulus to subcutaneous fat depots, including inguinal and interscapular adipose, but not visceral fat. At this point, we can only speculate on the disparate responses. This may be a result of the varying G protein-coupled receptor (GPCR) expression in adipose tissue depots [9]. GPCRs both respond to βHB [10] and stimulate mitochondrial uncoupling [11], making their differential adipose expression a possible explanation for our findings. 

When comparing the subcutaneous fat depots across humans and rats, the effect was similar, though the magnitude was greater in human adipose. This could be due to several reasons, including temperature variations (i.e., humans were likely occasionally exposed to colder temperatures) and inter-species differences. Namely, humans appear to have a more robust reliance on ketones for development, and even more rapidly and easily reach ketosis with diet [12,13]. 

The observation of significantly increased mitochondrial respiration in SAT in response to ketone exposure may play some part in the increased whole-body energy expenditure that has been seen in humans in ketosis. Most notably and recently, Ebbeling et al. [33] utilized doubly labeled water to determine the energy expenditure in free-living humans. By rotating study subjects across three diets varying in carbohydrates and fats, they found that energy expenditure was the greatest during adherence to the diet lowest in carbohydrates and highest in fat, a macronutrient profile that typifies a ketogenic diet. Furthermore, by scrutinizing energy expenditure in a metabolic ward, Hall et al. [34] found a similar result—energy expenditure was significantly elevated when the subjects followed a ketogenic diet. 

Type 1 diabetes presents an additional and relevant context to our findings. When untreated, the absence of insulin in type 1 diabetes results in unchecked lipolysis and ketogenesis, driving ketones to dangerously high levels, surpassing the plasma buffer capacity and impacting pH. Often, the weight loss of untreated type 1 diabetes is attributed to glucosuria—i.e., the loss of calories from excreted glucose causes weight loss. However, changes in urinary glucose excretion fail to correlate with the weight changes in type 1 diabetes [14]. Thus, to fully explain the dramatic weight changes in states of treated vs. untreated type 1 diabetes, additional explanation is needed. While renal glucose excretion certainly accounts for a loss of some glucose, and therefore a reduction in available calories, it does not explain the demonstrable differences in energy expenditure with the disease. Over 100 years ago, Joslin and Benedict noted that the metabolic rate in untreated insulin-deficient individuals with type 1 diabetes was roughly 15% higher compared with similar body weight subjects without type 1 diabetes [35]. Remarkably similar findings were observed decades later by Nair et al. [36]; energy expenditure was 20% higher than predicted in the absence of insulin therapy and, with the initiation of insulin, rapidly slowed to the predicted values. 

While insulin itself was the focus of the aforementioned studies into energy expenditure in type 1 diabetes, one cannot but wonder at the relevance of ketones; in other words, might some of the increased energy expenditure in states of low or deficient insulin be the result of ketone-induced mitochondrial uncoupling? Indeed, a confounding variable and essential consideration underlying our data and other studies that explore the adipocyte-specific, as well as whole-body, metabolic effects of ketones on energy expenditure and mitochondrial physiology is the hormone insulin. Insulin has direct and powerful control over ketogenesis. Briefly, a relative reduction in insulin disinhibits both adipocyte lipolysis and hepatic ketogenesis, thereby providing both substrate and stimulus for ketone production. Additionally, insulin dampens adipocyte mitochondrial uncoupling and, by extension, energy expenditure. We recently found that long-term insulin therapy to induce hyperinsulinemia in rodents was sufficient to reduce the whole-body energy expenditure [9]. Moreover, insulin inhibited mitochondrial uncoupling, resulting in more tightly coupled mitochondria in both BAT and SAT. Our future research efforts will explore the effect of elevated ketones alone, without the need for low basal insulin, on adipocyte mitochondrial uncoupling in vivo by using exogenous ketones. 

These results are thought-provoking when viewed through the lens of starvation. Food restriction is the most rapid stimulus for ketogenesis. Of course, in such a state of energy deprivation, it is difficult to imagine the body wasting energy through ketone-induced adipocyte mitochondrial uncoupling. Interestingly, short-term starvation (i.e., fasting) paradoxically increases energy expenditure. Zauner et al. [37] found that energy expenditure increased throughout the first two days of a four-day fast and remained elevated until the end. Coincidentally, plasma ketones followed a similar trend. However, the phenomenon has a limit—long-term starvation, such as that seen with anorexia nervosa, is associated with a reduction in the metabolic rate [38]. There are certainly other factors that mediate some of this effect, such as reduced muscle mass, but a relative insufficiency of adipose, and the lipid substrate for ketogenesis, may also be relevant.

This work adds a novel dimension to our previous findings regarding insulin’s effects on the adipocyte mitochondrial function [15]. Taken together, our observations that insulin enhances adipocyte mitochondrial coupling, while ketones drive uncoupling, may provide insight into obesity etiology. Specifically, these findings represent a unifying theory of two origins of obesity—calories and hormones. Elevated insulin and, thus, reduced ketones are an essential feature of human obesity [16,17,18,19]. In addition to inhibiting adipose lipolysis [20], insulin promotes both lipogenesis and adipogenesis in adipose tissue [21,22]. It is tempting to speculate that the contrasting changes in adipocyte mitochondrial respiration rates in states of varied insulin, with corresponding varied ketones, further contribute to adipocyte energy use and size and, by extension, obesity progression or regression. 

## 4. Materials and Methods

### 4.1. Cell Culture

3T3-L1 MBX murine fibroblast cells were maintained in Dulbecco’s modified Eagle’s medium (DMEM; D6546, Sigma-Aldrich, Saint Louis, MO, USA), plus 10% FBS (Invitrogen, Carlsbad, CA, USA) and 1% Penicillin-Streptomycin (P/S) (ThermoFisher, Waltham, MA, USA). The cells were grown in 10 cm culture dishes at 37 °C in a humidified atmosphere with 5% CO2. According to ATCC recommendations, the cells were strictly subcultured before they reached a density of 6 × 10^6^ viable cells/cm^2^. For differentiation into adipocytes, 3T3-L1 MBX fibroblasts were cultured to full confluency, followed by medium change with DMEM containing 10% fetal bovine serum (FBS, no. S 0615, Biochrom, Cambridge, UK), 1% P/S, 0.5 mM of 3-isobutyl-1-methylxanthine (IBMX), 0.25 μM of dexamethasone, 2 μM of rosiglitazone, and 1 μg/mL of insulin. After 48 h, the medium was changed to DMEM containing 10% FBS, 1% P/S, and 1 μg/mL of insulin for 48 h. On day 7, the medium was changed to DMEM containing 10% FBS and 1% P/S. This medium was refreshed on days 8, 10, 12, and 13. For the treatments, the cells were either incubated with water (for control) or βHB at 5 mM (54965, Sigma-Aldrich) for 24 h following the completion of the differentiation protocol. We elected to use 5 mM of βHB because this is both a level readily achievable in humans through diet and fasting [2] and one we have used previously [3].

### 4.2. Animals

All the animal procedures were approved (18-0101, approved 1-26-2018) by the institutional animal care and use committee at Brigham Young University. Twenty-week-old male and female Sprague-Dawley rats were housed for 1 week after arrival at the animal facility and were then provided with either a standard diet (SD; Envigo Teklad Rodent Diet, 8604; 32% protein, 14% fat, 54% carbohydrate) or a ketogenic diet (KD; Envigo Teklad custom diet; 22.4% protein, 77.1% fat (89% lard, 11% soybean oil), 0.5% carbohydrate) for 4 weeks. We elected to use lard as the primary fat over Crisco or soybean oil in light of evidence suggesting lard-based diets elicit better health in rodents [4]. Each day, the chow remaining in the cage was weighed and replaced with fresh chow. Body weight for the mice was recorded weekly. At 4 weeks, the rodents were euthanized, and blood and tissues were harvested. The plasma ketone levels were measured from a warmed tail vein using a Precision Xtra Ketone Monitor (Abbott, Columbus, OH, USA). Adipose not immediately used for respiratory analysis was snap frozen and stored at −80 °C for later analysis.

### 4.3. Mitochondrial Respiration

The cells and tissue were prepared for mitochondrial respiration, as described previously [5,14], before being transferred to respirometer chambers using the Oroboros O2K oxygraph (Oroboros, Innsbruck, Austria). Briefly, the cells were resuspended in MiR05 buffer (0.5 m ethylene glycol-bis-*N*,*N*,*N*′,*N*′-tetraacetic acid (EGTA)), 10 mM of KH_2_PO_4_, 3 mM of MgCl_2_·6H_2_O, 60 mM of K-lactobionate, 20 mM of Hepes, 110 mM of sucrose, and 1 mg/mL of fatty-acid-free bovine serum albumin (BSA; pH 7.1)) plus 1 mg/mL of digitonin and gently rocked at 37 °C for 5 min before transfer into respiration chambers. For adipose tissue, the samples were immediately placed in ice-cold buffer X (60 mM of K-Mes, 35 mM of KCl, 7.23 mM of K_2_-EGTA, 2.77 mM of CaK_2_-EGTA, 20 mM of imidazole, 20 mM of tuarine, 5.7 mM of ATP, 15 mM of PCr (phosphocreatine), and 6.56 mM of MgCl_2_·6H_2_O (pH 7.1)), and the connective tissue was removed. Following a 30 min incubation with 50 μg/mL of saponin and being rocked at 4 °C, the tissues were transferred to respiration chambers in MiR05. The electron flow through complex I was supported by glutamate + malate (10 mM and 2 mM, respectively; GM). Following stabilization, adenosine diphosphate (ADP; D) (2.5 mM) was added to determine the oxidative phosphorylation capacity. Succinate was added (S) for the complex I + II electron flow into the Q-junction. Preliminary assays tested the effect of carbonyl cyanide 4-(trifluoromethoxy) phenylhydrazone (FCCP; 0.05 μM) to test the full electron transport capacity, but this revealed no additive effect (data not shown). Cytochrome *c* (10 μM) was used to confirm the mitochondrial membrane integrity within each assay (data not shown). Basal respiration was determined through the addition of antimycin A (2.5 μM). The respiratory control ratio (RCR) was measured by the ratio of D:GM. Following the respiration protocol, the samples were removed from the chambers and used for further analysis, including ATP quantification. The ratio of ATP produced to peak oxygen consumed was used as an indicator of the coupling of oxygen transport to oxidative phosphorylation. The cell and tissue homogenates were analyzed for protein concentration using a modified Lowry assay (DC Protein Assay; Bio-Rad Laboratories, Hercules, CA, USA) according to the manufacturer’s protocols. 

### 4.4. Tissue Homogenization

Adipose samples were homogenized in ground-glass with lysis buffer (50 mM of Tris-HCl, pH 7.4; 250 mM of mannitol; 50 mM of NaF; 5 mM of sodium pyrophosphate; 1 mM of EDTA; 1 mM of EGTA; 1% Triton X-100; 50 mM of β-glycerophosphate; 1 mM of sodium orthovanadate; 1 mM of DTT; 1 mM of benzamidine; 0.1 mM of phenylmethane sulfonyl fluoride; 5 μg/mL of trypsin inhibitor), followed by centrifugation at 10,000× *g* for 10 min. 

### 4.5. ATP Measurements 

#### 4.5.1. Cell Culture

Following the PBS wash, the cells were trypsinized, pelleted, then lysed with 150 μL of 1 M perchloric acid on ice to precipitate cellular proteins. Following centrifugation at 20,000× *g* for 10 min, 150 μL of supernatant was transferred to a new tube with 150 μL of 1 M KOH. The ATP was measured with an ATP assay kit (Life Technologies, Carlsbad, CA, USA). 

#### 4.5.2. Tissue Samples

Rodent and human tissues were homogenized in 1 mL of lysis buffer using a glass pestle and mortar. For rodent tissue, the ATP was quantified following the snap freezing of fresh tissue. For human tissue, the ATP was quantified from tissue following the respiration protocol outlined above. Post-homogenization, 450 μL of each sample was aliquoted into a 96-well dish. ATP was measured with an ATP assay kit (Life Technologies, Carlsbad, CA, USA).

### 4.6. RT-qPCR

The total RNA was isolated from tissue samples using the RNeasy Lipid Tissue Mini Kit (QIAGEN) and measured on a NanoDrop 2000c spectrophotometer (Thermo Scientific). The mRNA in 2 μg of total RNA was converted to cDNA using oligo(dT) primer and random hexamers according to the manufacturer’s instructions (Clontech EcoDry Premix). The potential primers and probes were analyzed for the requirements imposed by real-time PCR using the PrimerQuest program. Finally, the chosen primers and probes were analyzed for specificity against GenBank sequences with the BLAST program package [39]. For thermal cycling and fluorescence detection, a LightCycler 480 machine was used. The threshold (*C*_t_) values of fluorescence for each readout were normalized to the corresponding *C*_t_ values [40], and the fold change was calculated using the ΔΔ*C*_t_ method [41].

### 4.7. Human Diet

Approval for human adipose biopsies was obtained by the Institutional Review Board at Brigham Young University (E-18334; approved 4-13-18). Female and male subjects were recruited based upon long-term self-adherence to either a ketogenic (KD; *n* = 5, 1 female, 4 male; age: 29.2 ± SEM 3.6; βHB: 1.8 ± 0.3 mmol/L; BMI: 25.5 ± 2.6 kg/m^2^) or standard American diet (SD; *n* = 4, 1 female, 3 male; age: 21.8 ± 0.5; βHB: 0.3 ± 0.1; BMI: 24.7 ± 0.6 kg/m^2^). Ketosis was confirmed by measuring the plasma ketones (Precision Xtra, Abbot, Columbus, OH, USA). 

### 4.8. Human Fat Biopsies

Percutaneous needle biopsies were taken from subcutaneous fat tissue near the navel. To perform the biopsy, a small area on the skin near the navel was shaved with an electric razor (when needed) and then cleaned with the antiseptic chlorhexidine. After sterilizing the area, the injection of a local anesthesia (1% lidocaine with epinephrine) was used for numbing. Upon the subject reporting no sensation in the area in response to gentle probing, a small incision (~1 cm) was made into the skin. Following this, the biopsy needle was inserted into the fat about 3 cm at a shallow (~30 degree) angle into the fat. Using manual suction, approximately 75 mg (about the size of a pencil eraser) of fat tissue was withdrawn. The sample was immediately placed in a cell buffer solution for the analysis of mitochondrial respiration. 

### 4.9. Statistical Methods

Data are presented as means ± SEM. The data were compared with the Student’s *t*-test (Graphpad Prism; Microsoft Excel). The data were considered statistically significant at *p* < 0.05.

## 5. Conclusions

In conclusion, these results indicate that ketones elicit a pronounced and perhaps even meaningful shift in mitochondrial function in adipose tissue. Whereas adipose tissue is generally a low metabolic rate organ that stores energy, ketones enable a fundamental and uncharacteristic shift towards energy wasting via the initiation of a futile cycle by increasing electron transport but not ATP synthesis. These findings shed light on previous observations of enhanced energy expenditure in ketogenic states, and may provide novel interventions in the future to help combat the growing trend of obesity and derivative disorders. 

## References

[1] Hales C.M., Carroll M.D., Fryar C.D., Ogden C.L. (2020). Prevalence of obesity and severe obesity among adults: United States, 2017–2018. NCHS Data Brief.

[2] Flegal K.M., Kruszon-Moran D., Carroll M.D., Fryar C.D., Ogden C.L. (2016). Trends in obesity among adults in the United States, 2005 to 2014. JAMA.

[3] Despres J. (1993). Abdominal obesity as important component of insulin-resistance syndrome. Nutrition.

[4] Bikman B.T. (2012). A role for sphingolipids in the pathophysiology of obesity-induced inflammation. Cell. Mol. Life Sci..

[5] Liu B., Page A.J., Hatzinikolas G., Chen M.X., Wittert G.A., Heilbronn L.K. (2019). Intermittent Fasting Improves Glucose Tolerance and Promotes Adipose Tissue Remodeling in Male Mice Fed a High-Fat Diet. Endocrinology.

[6] Calle E.E., Kaaks R. (2004). Overweight, obesity and cancer: Epidemiological evidence and proposed mechanisms. Nat. Rev. Cancer.

[7] Farmer S.R., Boss O. (2012). Recruitment of brown adipose tissue as a therapy for obesity-associated diseases. Front. Endocrinol..

[8] Cinti S. (2006). The role of brown adipose tissue in human obesity. Nutr. Metab. Cardiovasc. Dis..

[9] Dallon B.W., Parker B.A., Hodson A.E., Tippetts T.S., Harrison M.E., Appiah M.M.A., Witt J.E., Gibbs J.L., Gray H.M., Sant T.M. (2018). Insulin selectively reduces mitochondrial uncoupling in brown adipose tissue in mice. Biochem. J..

[10] Rosenbaum M., Leibel R.L. (2010). Adaptive thermogenesis in humans. Int. J. Obes..

[11] Choe S.S., Huh J.Y., Hwang I.J., Kim J.I., Kim J.B. (2016). Adipose tissue remodeling: Its role in energy metabolism and metabolic disorders. Front. Endocrinol..

[12] Bartelt A., Heeren J. (2014). Adipose tissue browning and metabolic health. Nat. Rev. Endocrinol..

[13] Gantner M.L., Hazen B.C., Conkright J., Kralli A. (2014). GADD45γ regulates the thermogenic capacity of brown adipose tissue. Proc. Natl. Acad. Sci. USA.

[14] Parker B.A., Walton C.M., Carr S.T., Andrus J.L., Cheung E.C., Duplisea M.J., Wilson E.K., Draney C., Lathen D.R., Kenner K.B. (2018). β-Hydroxybutyrate Elicits Favorable Mitochondrial Changes in Skeletal Muscle. Int. J. Mol. Sci..

[15] Youm Y.-H., Nguyen K.Y., Grant R.W., Goldberg E.L., Bodogai M., Kim D., D’agostino D., Planavsky N., Lupfer C., Kanneganti T.D. (2015). The ketone metabolite β-hydroxybutyrate blocks NLRP3 inflammasome–mediated inflammatory disease. Nat. Med..

[16] Frey S., Geffroy G., Desquiret-Dumas V., Gueguen N., Bris C., Belal S., Amati-Bonneau P., Chevrollier A., Barth M., Henrion D. (2017). The addition of ketone bodies alleviates mitochondrial dysfunction by restoring complex I assembly in a MELAS cellular model. Biochim. Biophys. Acta Mol. Basis Dis..

[17] Kim D.Y., Rho J.M. (2008). The ketogenic diet and epilepsy. Curr. Opin. Clin. Nutr. Metab. Care.

[18] Yuen A.W.C., Walcutt I.A., Sander J.W. (2017). An acidosis-sparing ketogenic (ASK) diet to improve efficacy and reduce adverse effects in the treatment of refractory epilepsy. Epilepsy Behav..

[19] Kim D.Y., Davis L.M., Sullivan P.G., Maalouf M., Simeone T.A., van Brederode J., Rho J.M. (2007). Ketone bodies are protective against oxidative stress in neocortical neurons. J. Neurochem..

[20] Myette-Cote E., Neudorf H., Rafiei H., Clarke K., Little J.P. (2018). Prior ingestion of exogenous ketone monoester attenuates the glycaemic response to an oral glucose tolerance test in healthy young individuals. J. Physiol..

[21] Seyfried B.T., Kiebish M., Marsh J., Mukherjee P. (2009). Targeting energy metabolism in brain cancer through calorie restriction and the ketogenic diet. J. Cancer Res. Ther..

[22] Cahill Jr G.F. (2006). Fuel metabolism in starvation. Annu. Rev. Nutr..

[23] Robinson A.M., Williamson D.H. (1980). Physiological roles of ketone bodies as substrates and signals in mammalian tissues. Physiol. Rev..

[24] McGarry J., Foster D. (1980). Regulation of hepatic fatty acid oxidation and ketone body production. Annu. Rev. Biochem..

[25] Rojas-Morales P., Tapia E., Pedraza-Chaverri J. (2016). β-hydroxybutyrate: A signaling metabolite in starvation response?. Cell. Signal..

[26] Kennedy A.R., Pissios P., Otu H., Roberson R., Xue B., Asakura K., Furukawa N., Marino F.E., Liu F.F., Kahn B.B. (2007). A high-fat, ketogenic diet induces a unique metabolic state in mice. Am. J. Physiol. Endocrinol. Metab..

[27] Hua Z.G., Xiong L.J., Yan C., Wei D.H., YingPai Z., Qing Z.Y., Lin Q.Z., Fei F.R., Ling W.Y., Ren M.Z. (2016). Glucose and Insulin Stimulate Lipogenesis in Porcine Adipocytes: Dissimilar and Identical Regulation Pathway for Key Transcription Factors. Mol. Cells.

[28] Kobayashi T., Fujimori K. (2012). Very long-chain-fatty acids enhance adipogenesis through coregulation of Elovl3 and PPARgamma in 3T3-L1 cells. Am. J. Physiol. Endocrinol. Metab..

[29] Srivastava S., Baxa U., Niu G., Chen X., Veech R.L. (2013). A ketogenic diet increases brown adipose tissue mitochondrial proteins and UCP1 levels in mice. IUBMB Life.

[30] Virtanen K.A., Lidell M.E., Orava J., Heglind M., Westergren R., Niemi T., Taittonen M., Laine J., Savisto N.J., Enerback S. (2009). Functional brown adipose tissue in healthy adults. N. Engl. J. Med..

[31] Vijgen G.H., Bouvy N.D., Teule G.J., Brans B., Schrauwen P., van Marken Lichtenbelt W.D. (2011). Brown adipose tissue in morbidly obese subjects. PLoS ONE.

[32] Kaisanlahti A., Glumoff T. (2019). Browning of white fat: Agents and implications for beige adipose tissue to type 2 diabetes. J. Physiol. Biochem..

[33] Ebbeling C.B., Feldman H.A., Klein G.L., Wong J.M.W., Bielak L., Steltz S.K., Luoto P.K., Wolfe R.R., Wong W.W., Ludwig D.S. (2018). Effects of a low carbohydrate diet on energy expenditure during weight loss maintenance: Randomized trial. BMJ.

[34] Hall K.D., Chen K.Y., Guo J., Lam Y.Y., Leibel R.L., Mayer L.E., Reitman M.L., Rosenbaum M., Smith S.R., Walsh B.T. (2016). Energy expenditure and body composition changes after an isocaloric ketogenic diet in overweight and obese men. Am. J. Clin. Nutr..

[35] Joslin EP B.F. (1912). A Study of Metabolism in Severe Diabetes.

[36] Nair K.S., Halliday D., Garrow J.S. (1984). Increased energy expenditure in poorly controlled Type 1 (insulin-dependent) diabetic patients. Diabetologia.

[37] Zauner C., Schneeweiss B., Kranz A., Madl C., Ratheiser K., Kramer L., Roth E., Schneider B., Lenz K. (2000). Resting energy expenditure in short-term starvation is increased as a result of an increase in serum norepinephrine. Am. J. Clin. Nutr..

[38] Melchior J.C., Rigaud D., Rozen R., Malon D., Apfelbaum M. (1989). Energy expenditure economy induced by decrease in lean body mass in anorexia nervosa. Eur. J. Clin. Nutr..

[39] Altschul S.F., Gish W., Miller W., Myers E.W., Lipman D.J. (1990). Basic local alignment search tool. J. Mol. Biol..

[40] Gong H., Sun L., Chen B., Han Y., Pang J., Wu W., Qi R., Zhang T.-M. (2016). Evaluation of candidate reference genes for RT-qPCR studies in three metabolism related tissues of mice after caloric restriction. Sci. Rep..

[41] Pfaffl M.W. (2001). A new mathematical model for relative quantification in real-time RT–PCR. Nucleic Acids Res..

